# Retraction Note to: Effect of Paclitaxel-Mesoporous Silica Nanoparticles with a Core-Shell Structure on the Human Lung Cancer Cell Line A549

**DOI:** 10.1186/s11671-022-03695-3

**Published:** 2022-06-07

**Authors:** Tieliang Wang, Ying Liu, Chao Wu

**Affiliations:** 1 Animal Husbandry and Veterinary Medicine School, Jinzhou Medical University, 40 Songpo Road, Linghe District, Jinzhou, Liaoning Province 121000 China; 2 Pharmacy School, Jinzhou Medical University, 40 Songpo Road, Linghe District, Jinzhou, Liaoning Province 121000 China

## Retraction Note: Nanoscale Res Lett (2017) 12:66 https://doi.org/10.1186/s11671-017-1826-1

The authors have retracted this article. After publication, it was noted that Figures 6 and 7 used incorrect data from a different study. This has led to the authors having a loss of confidence in the results of their article.

All authors agree to this retraction.

